# Mode of action of thiocoraline, a natural marine compound with anti-tumour activity

**DOI:** 10.1038/sj.bjc.6690451

**Published:** 1999-06

**Authors:** E Erba, D Bergamaschi, S Ronzoni, M Faretta, S Taverna, M Bonfanti, C V Catapano, G Faircloth, J Jimeno, M D'Incalci

**Affiliations:** Laboratory of Cancer Pharmacology, Department of Oncology, Istituto di Ricerche Farmacologiche ‘Mario Negri’, via Eritrea 62, Milano, 20157, Italy; Department of Experimental Oncology, Hollings Cancer Center, Medical University of South Carolina, 171 Ashley Avenue, Charleston, SC, 29425-2850, USA; PharmaMar USA Inc., 320 Putnam Avenue, Cambridge, MA, 02139-4616, USA; Research and Development, PharmaMar S.A., Calle de la Calera 3, Tres Cantos, Madrid, 28760, Spain

**Keywords:** natural compound, thiocoraline, cell cycle, DNA polymerase α

## Abstract

Thiocoraline, a new anticancer agent derived from the marine actinomycete *Micromonospora marina*, was found to induce profound perturbations of the cell cycle. On both LoVo and SW620 human colon cancer cell lines, thiocoraline caused an arrest in G1 phase of the cell cycle and a decrease in the rate of S phase progression towards G2/M phases, as assessed by using bromodeoxyuridine/DNA biparametric flow cytometric analysis. Thiocoraline does not inhibit DNA-topoisomerase II enzymes in vitro, nor does it induce DNA breakage in cells exposed to effective drug concentrations. The cell cycle effects observed after exposure to thiocoraline appear related to the inhibition of DNA replication. By using a primer extension assay it was found that thiocoraline inhibited DNA elongation by DNA polymerase α at concentrations that inhibited cell cycle progression and clonogenicity. These studies indicate that the new anticancer drug thiocoraline probably acts by inhibiting DNA polymerase α activity. © 1999 Cancer Research Campaign


*British Journal of Cancer* (1999) **80**(7), 971–980
© 1999 Cancer Research Campaign
Article no. bjoc.1998.0451

### Mode of action of thiocoraline, a natural marine compound with anti-tumour activity

**E Erba[1], D Bergamaschi[1], S Ronzoni[1], M Faretta[1], S Taverna[1], M Bonfanti[1], CV Catapano[2], G Faircloth[3], J Jimeno[4] and M D'Incalci[1]**

[1]Laboratory of Cancer Pharmacology, Department of Oncology, Istituto di Ricerche Farmacologiche 'Mario Negri', via Eritrea 62, 20157 Milano, Italy; [2]Department of Experimental Oncology, Hollings Cancer Center, Medical University of South Carolina, 171 Ashley Avenue, Charleston, SC 29425-2850, USA; [3]PharmaMar USA, Inc., 320 Putnam Avenue, Cambridge, MA 02139-4616, USA; [4]PharmaMar, S.A., Research and Development, Calle de la Calera 3, 28760 Tres Cantos, Madrid, Spain

**Summary** Thiocoraline, a new anticancer agent derived from the marine actinomycete *Micromonospora marina*, was found to induce profound perturbations of the cell cycle. On both LoVo and SW620 human colon cancer cell lines, thiocoraline caused an arrest in G1 phase of the cell cycle and a decrease in the rate of S phase progression towards G2/M phases, as assessed by using bromodeoxyuridine/DNA biparametric flow cytometric analysis. Thiocoraline does not inhibit DNA-topoisomerase II enzymes in vitro, nor does it induce DNA breakage in cells exposed to effective drug concentrations. The cell cycle effects observed after exposure to thiocoraline appear related to the inhibition of DNA replication. By using a primer extension assay it was found that thiocoraline inhibited DNA elongation by DNA polymerase α at concentrations that inhibited cell cycle progression and clonogenicity. These studies indicate that the new anticancer drug thiocoraline probably acts by inhibiting DNA polymerase α activity.

**Keywords:** natural compound; thiocoraline; cell cycle; DNA polymerase α

Thiocoraline is a thiodepsipeptide, isolated from an actinomycete, the *Micromonospora marina*, a microorganism collected off the Mozambique coast (Perez Baz et al, 1997; Romero et al, 1997). It has shown antiproliferative activity against human non-small cell lung, breast, colon, renal and melanoma cancer cell type subpanels in the National Cancer Institute in vitro screening program. Subsequently, thiocoraline also exhibited anti-tumour activity in vivo against human carcinoma xenografts (Faircloth et al, 1997).

The lack of information on the mechanism of the antiproliferative and anti-tumour activity of thiocoraline prompted us to perform this study aimed at evaluating the cell cycle perturbations induced by this novel drug in human cancer cell lines and elucidating its biochemical mode of action.

### MATERIALS AND METHODS

### Cells and culture conditions

The human colon adenocarcinoma LoVo cell line was grown in monolayer in Ham's F12 medium supplemented with 10% fetal calf serum, 1% vitamins (BME vitamin solution, 100×), 1% glutamine 200 mM (Gibco Europe, Paisley, UK) at 37°C in a humidified 5% carbon dioxide atmosphere in T25 $cm^2$ tissue flasks (IWAKI, Bibby Sterilin, Staffordshire, UK). The human colon adenocarcinoma SW620 cell line was grown in monolayer in RPMI-1640 medium supplemented with 10% fetal calf serum in T25 $cm^2$ tissue flasks.

*Received 7 July 1998*
*Revised 19 November 1998*
*Accepted 27 November 1998*

*Correspondence to:* E Erba

### Clonogenicity test

Thiocoraline (Figure 1) was kindly supplied by Pharma Mar (S.A. Tres Cantos, Spain). The effect of the thiocoraline treatment on LoVo and SW620 cells was evaluated by a standard clonogenic assay. Exponentially growing cells were treated for 1, 6 or 24 h with different concentrations of thiocoraline. After treatment, cells were washed twice with phosphate buffered saline (PBS); 800 control and treated cells were plated in 30-mm Petri dishes with 3 ml fresh medium. Cell viability was checked using erythrosin B. The colonies were allowed to develop for 10–14 days. Plating efficiency of the exponentially growing control cells was between 50 and 60%. The colonies were stained with 1% crystal violet solution in 20% ethanol, and the number of colonies were measured using the Entry Level image system (Immagini & Computer, Bareggio, Milan, Italia). A background correction was made, and the smallest control cell colony was taken as the minimum for setting the cut-off point (Erba et al, 1994).

### Bromodeoxyuridine incorporation

This protocol allowed us to study and compare the DNA synthesis rate and labelling index (percentage of cells in synthetic phase that are labelled with bromodeoxyuridine (BrdU)) at different times after thiocoraline treatment and drug-washout. Exponentially growing LoVo and SW620 cells were treated for 24 h with 0.5 μM thiocoraline, which caused 30–40% growth inhibition at 24 h after drug-washout. After treatment, the drug-containing medium was removed, cells were washed twice with PBS and fresh medium was provided. During the last 15 min drug treatment, and at 24, 48 and 72 h after drug-washout, 20 μM BrdU was added to the cells, then the cells were directly fixed in 70% ethanol.

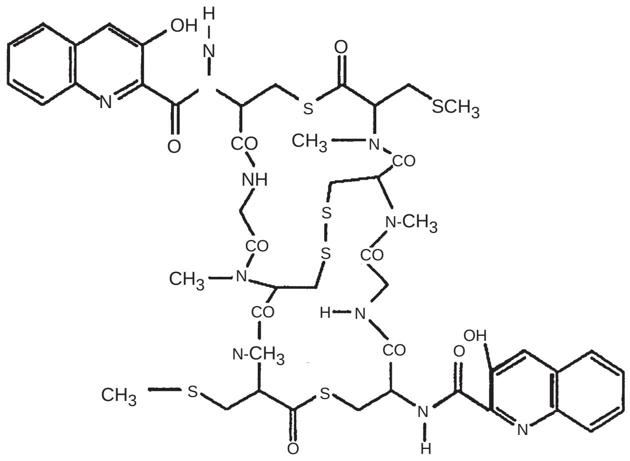

**Figure 1** Thiocoraline chemical structure

### Cell kinetic studies

With this protocol it was possible to obtain a distinct evaluation of the cell cycle perturbations in cells that were in S phase (BrdU-positive cells) or in G1 or in G2/M phases (BrdU-negative cells) when thiocoraline treatment started. Exponentially growing cells were treated for 15 min with 20 μM BrdU. At the end of BrdU incubation the medium was removed, cells were washed twice with PBS and treated for 24 h with 0.5 μM thiocoraline. After treatment the drug-containing medium was removed, the cells were washed twice with PBS and fresh medium was provided. At 0, 4, 8, 12 and 24 h during drug treatment, and 4, 8, 12, 24 h after drug-washout, control and treated cells were fixed in 70% ethanol and kept at 4°C before staining.

### BrdU/DNA staining

For detection of BrdU incorporation into DNA, the fixed cells were washed with cold PBS and the DNA was denatured with 1 ml of 3 N hydrochloric acid for 20 min at room temperature to allow the anti-BrdU monoclonal antibody (mAb) to react with the BrdU incorporated in the DNA chain. DNA denaturation was stopped by adding 3 ml of 0.1 M sodium tetraborate pH 8.5. After centrifugation, the pellet was incubated with 1 ml of 0.5% Tween-20 (Sigma Chemicals Co., St Louis, MO, USA) in PBS and 1% normal goat serum (NGS) (Dakopatts, Denmark) for 15 min at room temperature. The incorporated BrdU was visualized by incubating the cells with the mAb anti-BrdU (Becton Dickinson, Sunnyvale, CA, USA) diluted 1:10 in 0.5% Tween-20 in PBS for 1 h at room temperature in the dark. After centrifugation, the pellet was incubated with 1 ml of 0.5% Tween-20 and 1% NGS for 15 min at room temperature. Then the cells were incubated with a fluorescein isothiocyanate (FITC)-conjugated affinipure F(ab′)2 fragment goat anti-mouse IgG (Jackson Immuno Research Laboratories Inc, USA) diluted 1:50 in 0.5% Tween-20 in PBS for 1 h at room temperature in the dark. The cells were finally resuspended in 2 ml of a solution containing 2.5 μg $ml^{-1}$ of propidium iodide (PI) in PBS and 25 μl RNAase 1 mg $ml^{-1}$ in water, and stained overnight at 4°C in the dark. Monoparametric DNA and biparametric BrdU/DNA analysis was performed on at least 20 000 cells for each sample by the FacSort system (Becton Dickinson). The data are the average of three replications and were analysed with the Cell Quest software. The fluorescence pulses were detected using a band pass filter 530 ± 30 nm and 620 ± 35 nm, for green and red fluorescence respectively, in combination with a dichroic mirror 570 nm (Erba et al, 1995). The percentage of the cell cycle phase distribution were calculated by the method of Krishan and Frei (Krishan and Frei, 1976).

### Cyclin D1/DNA staining

At the end of treatment, and at different time intervals after drug washout, the cells were fixed in 1% formaldehyde and stored at 4°C. The fixed cells were washed in cold PBS and permeabilized with 0.25% Triton X-100 (Sigma, St Louis, MO, USA) in PBS for 5 min in ice. Then they were washed in PBS and incubated with 200 μl of mAb anti-human cyclin D1, G124-326 clone (Pharmingen, San Diego, CA, USA), at the concentration of 2.5 μg $ml^{-1}$ in PBS + 1% NGS overnight at 4°C in the dark. A blank sample was prepared by incubation of cells with 200 μl of 1% NGS in PBS, or with the isotype IgG instead of the cyclin.

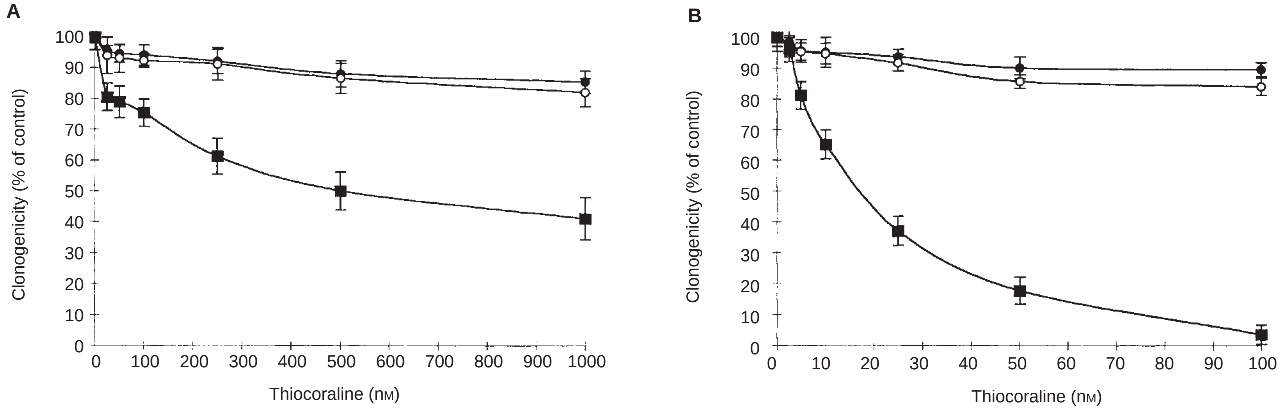

**Figure 2** Effect of thiocoraline on the clonogenicity of LoVo and SW620 cells. Clonogenic potential of exponentially growing untreated cells ranged between 50 and 60% of the cells plated, which was normalized to 100%. Each point is the mean of six replicates; bar, standard error of the mean. (●) = 1 h treatment; (○) = 6 h treatment; (■) = 24 h treatment. (**A**) LoVo cells; (**B**) SW620 cells

*© 1999 Cancer Research Campaign*

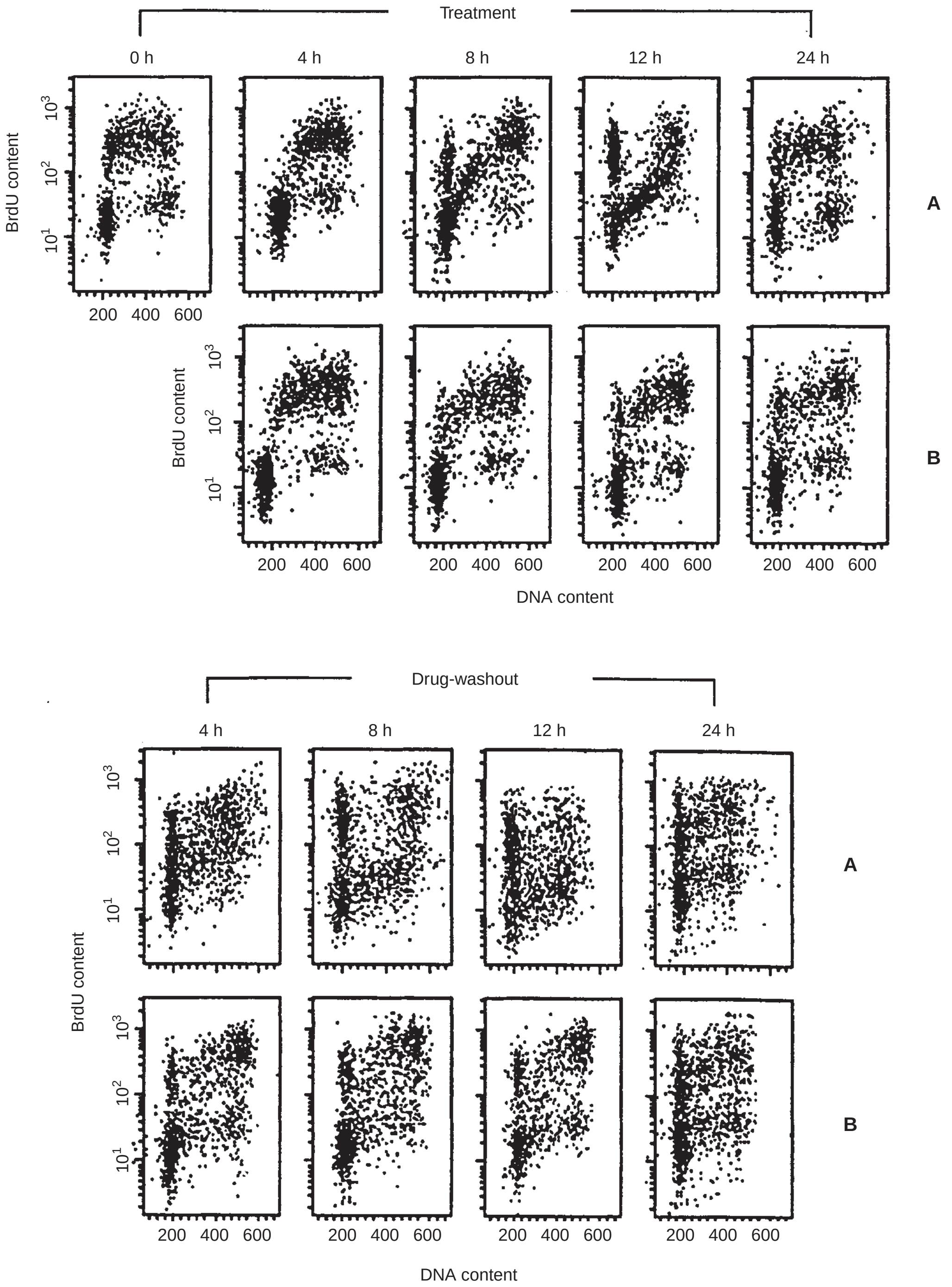

**Figure 3** Effects of thiocoraline on the cell cycle phase distribution of LoVo cells, 0, 4, 8, 12 and 24 h during drug treatment, and 4, 8, 12 and 24 h after drug-washout. *Upper panel*: (**A**) Biparametric BrdU/DNA analysis of control cells. (**B**) Biparametric BrdU/DNA analysis of thiocoraline-treated cells. *Lower panel*: (**A**) DNA histograms of control cells (whole population). (**B**) DNA histograms of thiocoraline-treated cells (whole population). (**C**) DNA histograms of BrdU-negative control cells. (**D**) DNA histograms of BrdU-negative thiocoraline-treated cells. (**E**) DNA histograms of BrdU-positive control cells. (**F**) DNA histograms of BrdU-positive thiocoraline-treated cells

After removing the cyclin D1, the cells were incubated with 200 μl of FITC-conjugated affinipure F(ab′)2 fragment goat anti-mouse IgG diluted 1:50 in 0.5% Tween-20 in PBS for 1 h at room temperature in the dark. The cells were finally resuspended in 2 ml of a solution containing 2.5 μg $ml^{-1}$ PI in PBS and 25 μl RNAase 1 mg $ml^{-1}$ in water, and stained overnight at 4°C in the dark (Faretta et al, 1998).

### Alkaline elution

LoVo and SW620 cells were labelled for 24 h using medium supplemented with 0.5 μCi $ml^{-1}$ $^3$H-TdR (Amersham, specific activity 85 Ci $mmol^{-1}$) and exposed to different concentrations of thiocoraline for 24 h. Then the cells were kept in ice, washed with PBS and layered on polycarbonate filters, 2-μm pore size and 25-mm diameter

© 1999 Cancer Research Campaign 

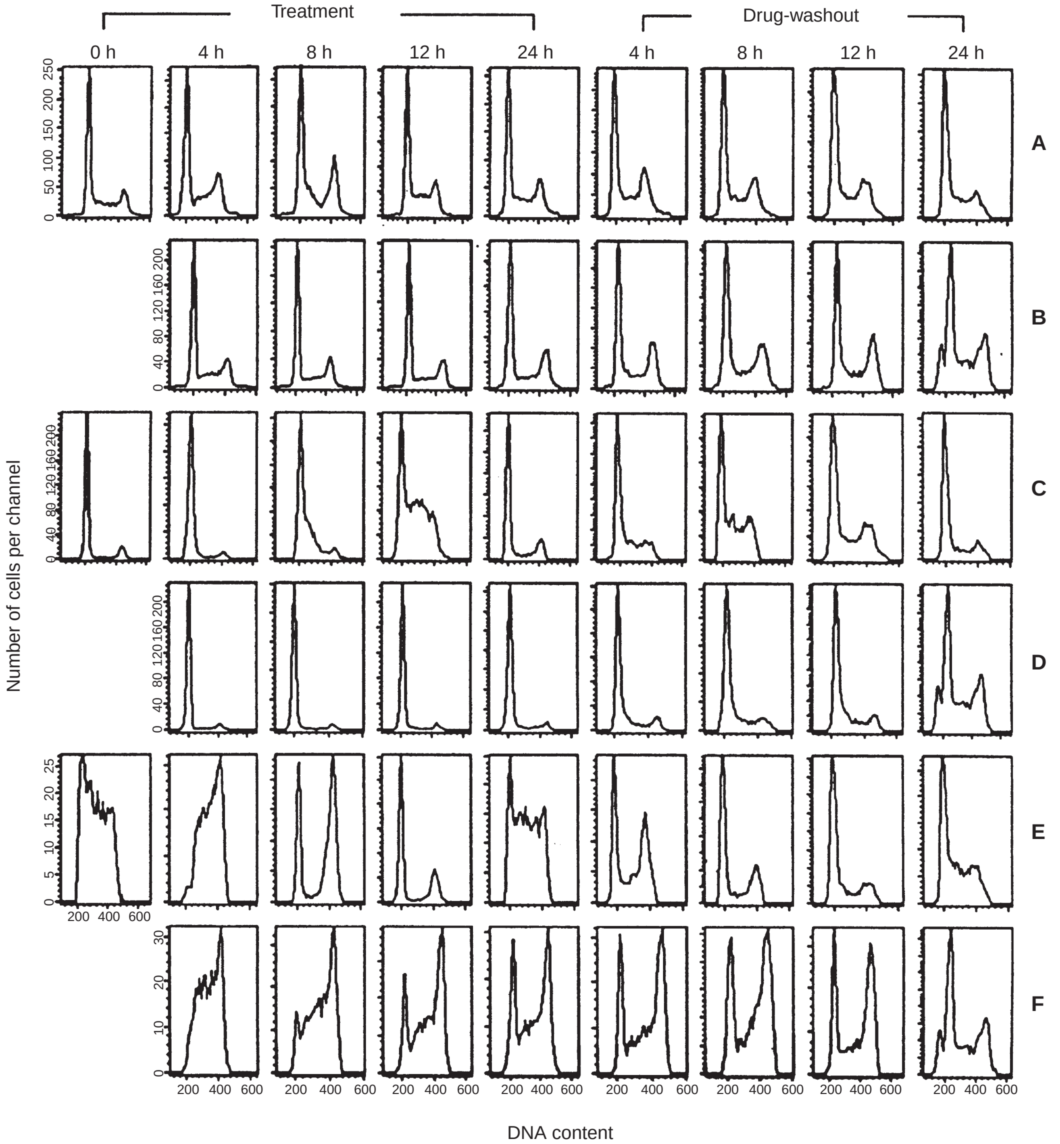

**Figure 3** *cont.*

(Nucleopore, Costar). Cells ($10^6$ cells per sample) were lysed with 5 ml of a lysis solution containing 2% sodium dodecyl sulphate (SDS), 0.02 M EDTA, 0.1 M glycine (pH 10), which was allowed to flow through the filter by gravity. After connecting the outlet of the filter holders to the pumping system, 2 ml of the lysis solution containing 0.5 mg $ml^{-1}$ proteinase K (Merk) were added to the reservoir over the polycarbonate filters. DNA was eluted from the filters with a solution containing 20 mM EDTA, 0.1% SDS and adjusted to pH 12.2 with tetrapropylammonium hydroxide (Fluka). Three-, 6-, 9-, 12- and 15-h fractions were collected, and fraction and filters were processed as previously described (Kohn et al, 1981).

### DNA unwinding assay

The assay was performed by using a 20 µl final reaction volume containing supercoiled PUC18 DNA, 50 mM Tris, pH 8, 120 mM potassium chloride (KCl), 10 mM-magnesium chloride ($MgCl_2$), 0.5 mM adenosine 5′-triphosphate (ATP), 0.5 mM dithiothreitol (DTT), 30 µg $ml^{-1}$ nuclease free bovine serum albumin (BSA), different concentrations of thiocoraline and 2 units of human type II topoisomerase (topoGEN). As positive control we used 125 µM m-AMSA (purchased from Sigma). Reactions were performed at 37°C for 30 min and stopped by addition of stop buffer (5% sarcosyl, 0.0025% bromophenol blue, 25% glycerol). Gel analysis was performed on 1.5% agarose gel. Gel was stained with ethidium bromide, washed with distilled water and photographed under UV light.

### Cleavable complex formation

The assay was performed as described by Poddevin et al (1993), with slight modification. Briefly, a 30 µl final reaction volume containing 25 ng of 5′-end $^{32}P$-labelled SV40 DNA (H*pa*II, *Ban*I), 10 mM Tris, pH 7.5, 50 mM KCl, 5 mM $MgCl_2$, 0.1 mM EDTA, 0.5 mM DTT, 1 mM ATP, 3 units of human topoGEN, different concentrations of thiocoraline were used. As positive control we used 0.5 µM doxorubicin (kindly donated by Dr Suarato, Pharmacia Upjohn). Reactions were performed at 37°C for 30 min and stopped

*© 1999 Cancer Research Campaign*

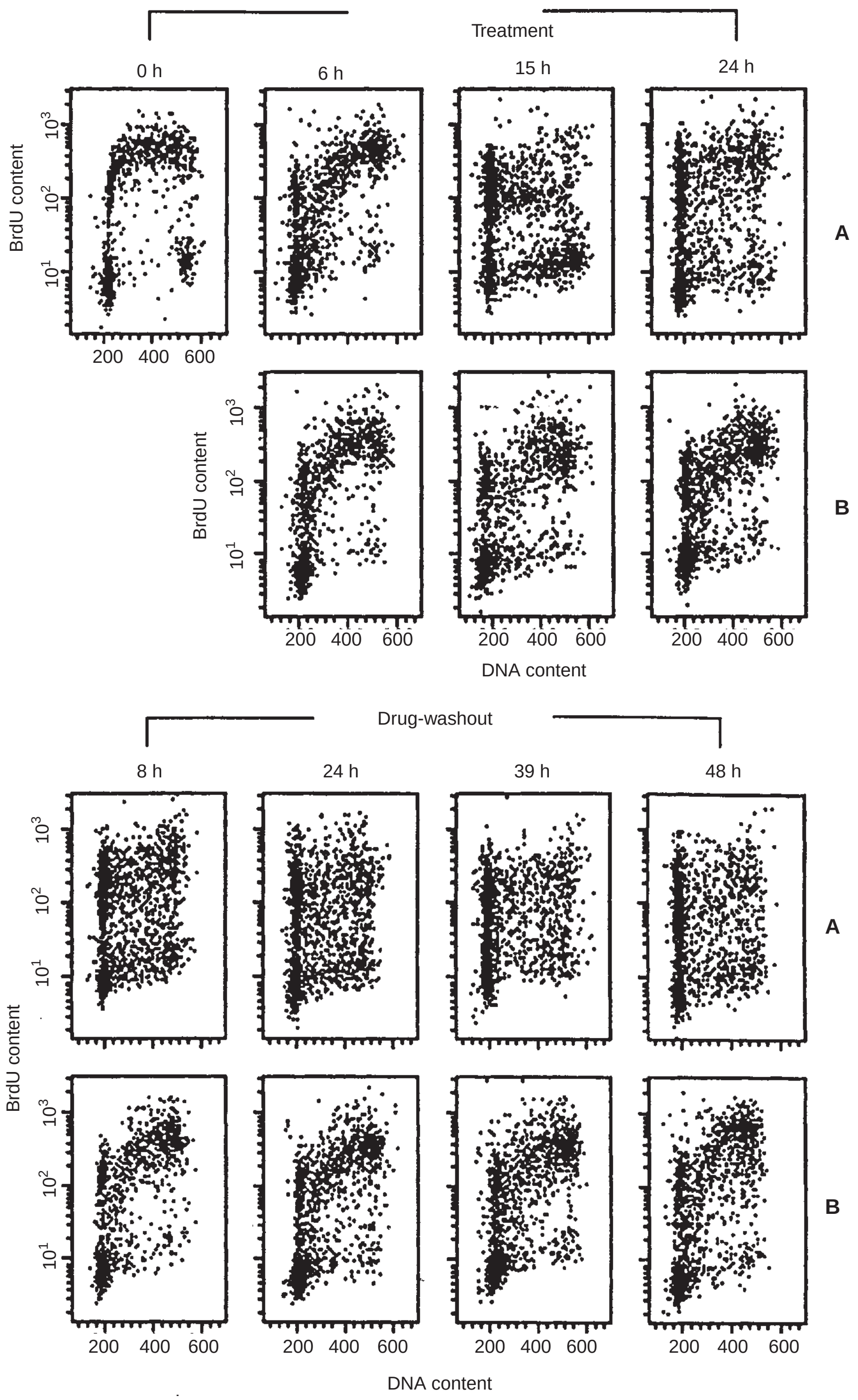

**Figure 4** Effects of thiocoraline on the cell cycle phase distribution of SW620 cells, 0, 6, 15 and 24 h during drug treatment, and 8, 24, 39 and 48 h after drug-washout. *Upper panel*: (**A**) Biparametric BrdU/DNA analysis of control cells. (**B**) Biparametric BrdU/DNA analysis of thiocoraline-treated cells. *Lower panel*: (**A**) DNA histograms of control cells (whole population). (**B**) DNA histograms of thiocoraline-treated cells (whole population). (**C**) DNA histograms of BrdU-negative control cells. (**D**) DNA histograms of BrdU-negative thiocoraline-treated cells. (**E**) DNA histograms of BrdU-positive control cells. (**F**) DNA histograms of BrdU-positive thiocoraline-treated cells by addition of 3 μl of SDS 10% and 3 μl of proteinase K 5 mg $ml^{-1}$, followed by incubation for 40 min at 42°C. After phenol–cloroform extraction 6 μl of loading buffer (5% sarkosyl, 0.0025% bromophenol blue, 25% glycerol) were added to each sample before loading onto 1% agarose gel made in 1 × Tris–borate–EDTA buffer plus 0.1% SDS. Gel was dried and autoradiographed for 12 h.

### DNA polymerase assay

Human DNA polymerase α was purified from acute lymphoblastic leukaemia CCRF-CEM cells by immunoaffinity chromatography (Catapano et al, 1993). Two oligodeoxyribonucleotides, the 11-mer 5′-GAATTCAGATC-3′ and the 25-mer 5′-GCGGTGACCCGGGA-

*© 1999 Cancer Research Campaign*

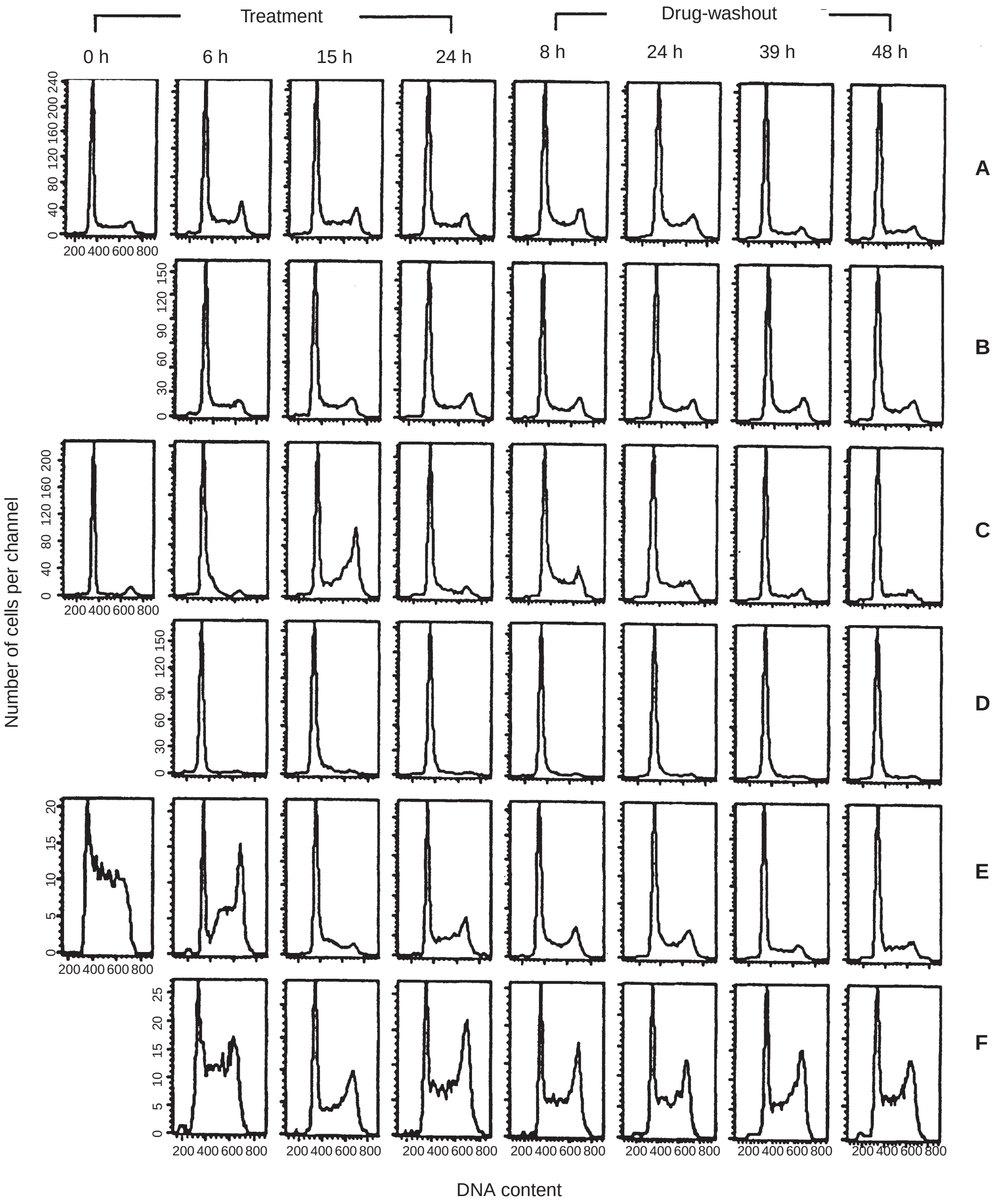

**Figure 4** *cont.*

GATCTGAATTC-3′, were synthesized. Prior to the primer extension assay, the 11-mer and 25-mer were annealed by heating at 90°C for 5 min followed by cooling to room temperature over a period of 1 h. Primer extension reactions (10 µl) contained 1 µM of the annealed 11- and 25-mers, 0.75 units of DNA polymerase α, 10 µM dNTPs with 1 µCi of [α-$^{32}$P]dCP (20 000 dpm $pmol^{-1}$), 10 mM $MgCl_2$, 100 µg $ml^{-1}$ of BSA, 1 mM DTT, 50 mM Tris–HCl pH 7.5, and the indicated concentrations of either dimethyl sulphoxide (DMSO) or thiocoraline. The samples were incubated for 60 min at 37°C. The reactions were then stopped by addition of EDTA and loading buffered, and electrophoresed in a denaturating 20% polyacrylamide gel as previously described. The products of the primer extension reactions were measured by scanning the gel with a phosphoimager and analysing the digitized images with the ImageQuant software (Molecular Dynamics). The counts measured in a sample lacking the enzyme (Figure 9, lane 8) were subtracted from the radioactivity measured in the complete reactions. The results were expressed as percentage of products synthesized in the presence of thiocoraline compared to the control reaction lacking both DMSO and thiocoraline (Figure 10, lane 1).

### RESULTS

Figure 2 shows the clonogenic inhibitory effect of thiocoraline on LoVo (Figure 2A) and SW620 cells (Figure 2B). Exposure for 1 or 6 h with thiocoraline did not cause a significant inhibition in the clonogenicity of SW620 and LoVo cells. However, a 24 h exposure to thiocoraline resulted in a much higher cytotoxicity on both cell lines. SW620 cells were more sensitive to thiocoraline than LoVo cells, with an $IC_{50}$ of 15 nM and 500 nM respectively.

 *© 1999 Cancer Research Campaign*

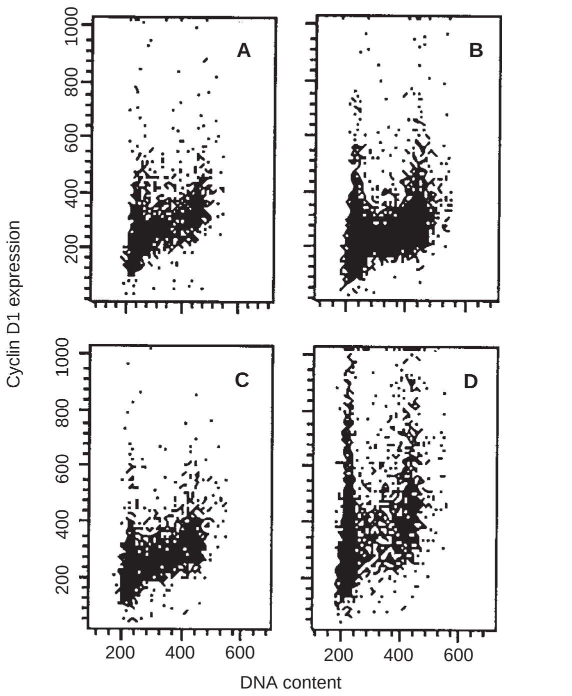

**Figure 5** Biparametric cyclin D1/DNA flow cytometric analysis of LoVo cells treated with 0.5 μM thiocoraline evaluated at 24 h drug treatment (**A**, control cells; **B**, thiocoraline-treated cells) and at 24 h after drug-washout (**C**, control cells; **D**, thiocoraline-treated cells)

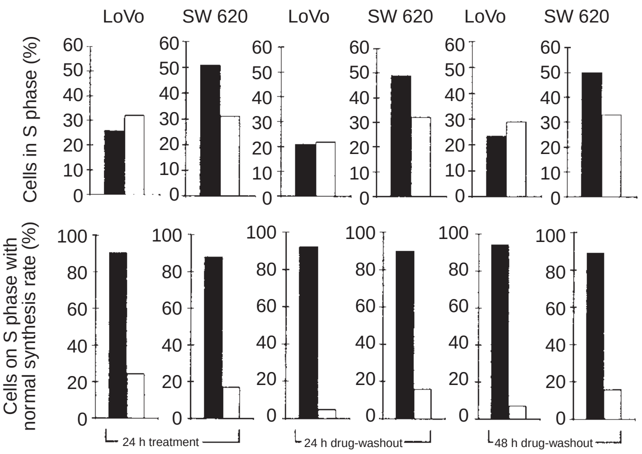

**Figure 6** Effects on DNA synthesis caused by thiocoraline exposure on LoVo and SW620 cells evaluated by BrdU/DNA analysis. During the last 15 min thiocoraline treatment, and at 24, 48 and 72 h after drug-washout, 20 μM BrdU were added to the cells, then the cells were directly fixed in 70% ethanol and kept at 4°C in the dark. (■) = control cells; (□) = thiocoraline-treated cells

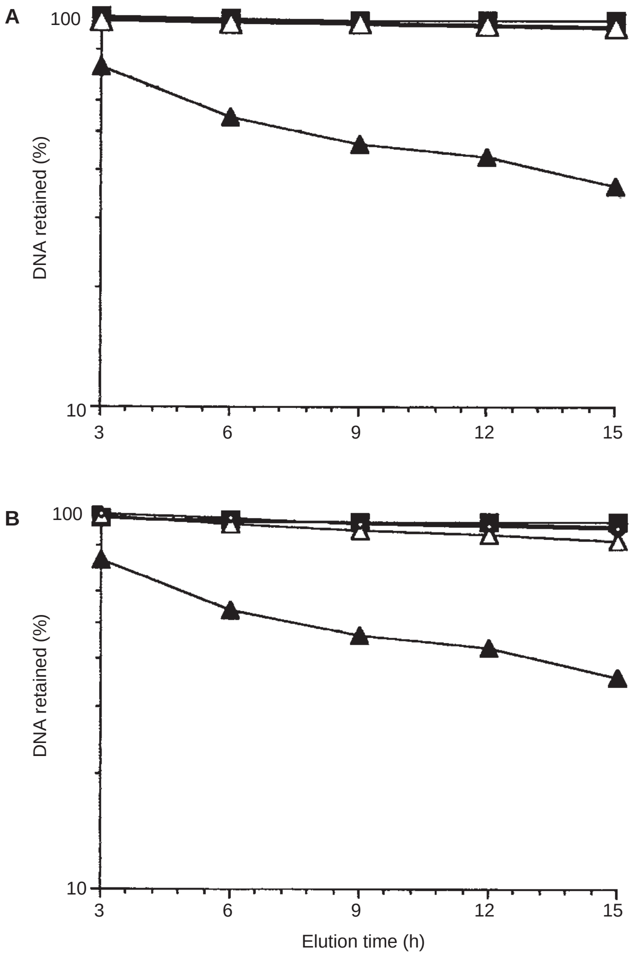

**Figure 7** Formation of DNA breaks on LoVo and SW620 cells exposed to different concentrations of thiocoraline for 24 h. (**A**) LoVo cells; (**B**) SW620 cells. (■) = control cells; (▲) = control cells irradiated at the dose of 150 rads; (●) = 0.5 μM thiocoraline; (◇) = 1 μM thiocoraline; (△) = 5 μM thiocoraline

The cell cycle phase perturbations induced by thiocoraline on LoVo and SW620 cells were investigated by monoparametric flow cytometric DNA analysis after 24 h treatment and at 24 h after drug-washout. By using PI staining, the effects of thiocoraline on the cell cycle phase distribution was not evident, since the DNA histograms of thiocoraline-treated cells were similar to the controls (data not shown). For this reason, we performed a biparametric flow cytometric BrdU/DNA analysis to evaluate the effect of thiocoraline exposure on cell cycle phase distributions, considered separately the BrdU+ (S phase) and the BrdU – (G1 or G2/M phases) cells. Figures 3 and 4 show the effects on the cell cycle phase distributions caused by thiocoraline on LoVo (Figure 3) and on SW620 cell line (Figure 4) during drug treatment and after drug-washout, evaluated using the biparametric BrdU/DNA analysis. The upper panel shows the biparametric analysis of BrdU and DNA content, while the lower panel separately shows the DNA histograms of the whole population (A and B), the DNA histograms of the BrdU – (C and D) and the BrdU+ cells (E and F). The thiocoraline-induced cell cycle perturbations were similar in LoVo and SW620 cell lines. Thiocoraline was found to prevent those cells that were in G1 when drug treatment started (BrdU – cells) from entering S phase. At 24 h drug treatment BrdU – SW620 cells were still completely blocked in G1 phase, whereas a small fraction of BrdU – LoVo cells progressed to S-late G2/M phases.

The BrdU+ cells, those cells which were in S phase when thiocoraline treatment started, were delayed in their progression from the S phase into the G2/M phase. Between 8 and 24 h for LoVo, and between 15 and 24 h for the SW620 cell line the BrdU+ thiocoraline-treated cells remained accumulated in the S and the G2/M phases, while the control cells have started a new cell cycle (G1

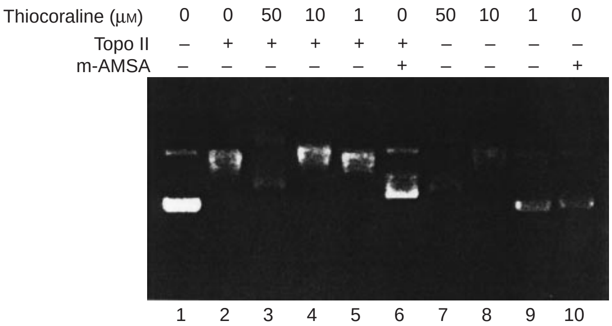

**Figure 8** DNA unwinding assay. Supercoiled pUC18 DNA was incubated with 50, 10 and 1 µM thiocoraline in absence, or in presence, of 2 units of human topoisomerase II, as described in Materials and Methods. As positive control we used 125 µM m-AMSA

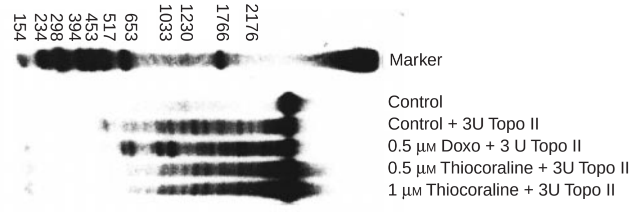

**Figure 9** Cleavable complex formation. 5′-end $^{32}$P-labelled SV40 DNA was incubated with 0.5 or 1 µM thiocoraline in presence of 3 units of human topoisomerase II (Topo II), as described in Materials and Methods. As positive control we used 0.5 µM doxorubicin (Doxo)

**A**

5′-GAATTCAGATC-3′
3′-CTTAAGTCTAGAGGGCCCAGTGGCG-5′

**B**

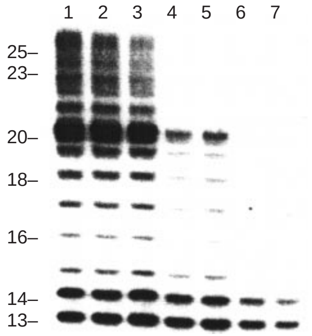

**Figure 10** Effects of thiocoraline treatment on the DNA polymerase α activity. (**A**) Sequence of the 11-mer primer and 25-mer template DNA used in the polymerase assay. (**B**) Autoradiography of a denaturating polyacrylamide gel. Primer extension reactions containing various concentrations of thiocoraline were incubated at 37°C for 60 min, denaturated by heating at 90°C for 5 min, and then loaded on the 20% polyacrylamide gel. Complete reaction (lane 1) contained 0.75 units of DNA polymerase α, 1 µM of primer/template, 10 µM dNTPs and 1 µCi of (α-$^{32}$P)dCTP. Samples in lanes 2–7 had the following additions: lane 2, 0.001% DMSO; lane 3, 0.01% DMSO; lane 4, 0.01 µM thiocoraline and 0.0001% DMSO; lane 5, 0.05 µM thiocoraline and 0.005% DMSO; lane 6, 0.1 µM thiocoraline and 0.001% DMSO; lane 7, 1 µM thiocoraline and 0.01% DMSO. The sample in lane 8 had all the components of the complete reaction minus the enzyme. Numbers to the left of lane 1 indicate the size in nucleotides of the corresponding primer extension products

BrdU+ cells). At 24 h after drug-washout, the BrdU+ and BrdU – LoVo cells started to cycle again, while the SW620 cells did not reverse their thiocoraline-induced cell cycle perturbations.

A biparametric cyclin D1/DNA analysis was then performed to characterize the cell cycle phase perturbations induced by thiocoraline more precisely. Figure 5 shows the levels of cyclin D1 in LoVo cells after 24 h thiocoraline treatment and after drug-washout. The level of cyclin D1 was not changed as compared to control cells, thus excluding the possibility that the slow progression of thiocoraline-treated cells through the cell cycle phase was due to changes in the level of this cyclin. The level of cyclin D1 increased at 24 h after drug-washout when the cells moved from G1 to S phase of the cell cycle. Similar increase in cyclin D1 level was obtained also on SW620 cells. Furthermore, thiocoraline did not cause an inhibition of cyclin D1-associated kinase activity (data not shown).

Figure 6 shows the effects on DNA synthesis caused by thiocoraline exposure. Thiocoraline did not cause a decrease in the fraction of LoVo cells in the S phase of the cell cycle, while their capacity to synthesize DNA was almost completely arrested after 24 h treatment and up to 48 h drug-washout. In SW620 cells, thiocoraline caused nearly a 50% decrease in the number of cells in the S phase of the cell cycle. Their capacity to synthetize DNA was reduced and this effect was evident after 24 h treatment and up to 48 h drug-washout.

Figure 7 shows the results of the experiment performed to evaluate if thiocoraline was able to cause DNA breaks on LoVo and on SW620 cells by using the alkaline elution method. DNA of thiocoraline-treated cells was almost completely retained on filter like the DNA of drug-untreated cells, whereas, as expected, a large fraction of DNA of irradiated cells was eluted through the filter. These results indicate that 24 h thiocoraline treatment did not cause detectable DNA breaks at the concentrations that inhibited growth rate of 50%.

Using topoGen unwinding assay we evaluated the inhibition of topoGen catalytic activity by thiocoraline. As shown in Figure 8, supercoiled PUC18 DNA (lane 1) was relaxed by Topoisomerase II in the absence (lane 2) or presence of 50, 10 and 1 µM thiocoraline (lanes 3, 4 and 5 respectively). Plasmid was also incubated with the same concentrations of thiocoraline in the absence of Topoisomerase II (lanes 7, 8 and 9). With thiocoraline we obtained the same results, in terms of DNA unwinding, both in presence or absence of topoGen enzyme (compare lanes 3 and 4 with 7 and 8). A concentration of 1 µM thiocoraline seemed to be ineffective to exert DNA relaxation in absence of topoGen. In the presence of Topoisomerase II, m-AMSA, used as a positive control, caused DNA unwinding (lanes 6).

These results would indicate that thiocoraline is not acting as a Topoisomerase II inhibitor. Its ability to cause unwinding of DNA is likely due to its binding to DNA, as already reported for several intercalating agents. Several DNA topoisomerase inhibitors have been reported to act as trapping cleavable complexes formed between the enzyme and the DNA. Stabilization of the cleavable complex results in single- or double-stranded DNA cleavage, which can be assessed by using a sensitive technique based on the detection of drug-induced DNA double-strand breaks in linearized 5′-end $^{32}$P-labelled SV40 DNA. Figure 9 shows that a pattern of cleavage sites can be observed on the labelled DNA incubated with Topoisomerase II (compare lanes 1 and 2) and this pattern was not altered when thiocoraline was added to the reaction mixture at concentrations of 0.5 and 1 µM (lanes 4 and 5). Doxorubicin, a known DNA Topoisomerase II inhibitor, was used

 *© 1999 Cancer Research Campaign*

as positive control. 0.5 μM doxorubicin was able to induce topoGen-mediated cleavage, as shown in Figure 9 (lane 3). These results show that thiocoraline does not act as a DNA topoGen inhibitor.

The effects of thiocoraline on DNA polymerase activity were evaluated using the DNA primer extension assay (Figure 10). In this assay, DNA polymerase α catalysed the polymerization of dNTPs onto an 11-mer DNA primer annealed to a 25-mer template (Figure 10A). In the absence of the inhibitor, DNA polymerase α produced a ladder of DNA fragments from 13 to 25 nucleotides in length (Figure 10B, lane 1). The 13-mer was the shortest product detected by autoradiography since the first radiolabelled dCMP residue was incorporated opposite the guanine at position 13 in the template. The addition of concentrations of DMSO (0.001 and 0.01% in lanes 2 and 3 respectively), similar to those present in the samples containing thiocoraline, did not affect DNA polymerase α activity. Primer extension was greatly inhibited in the presence of concentrations of thiocoraline from 0.01 to 1 μM (Figure 10B, lanes 4–7). The total amount of extended primers (i.e. all the products ≥ 13 nucleotides long) was reduced more than 60 and 80% by 0.01 and 0.1 μM of thiocoraline respectively. We also analysed the effects of thiocoraline on the synthesis of products that were either < 20 or ≥ 20 nucleotides long using the ImageQuant software. This analysis indicated that the synthesis of products ≥ 20 nucleotides long was almost completely inhibited by thiocoraline (83 and 93% inhibition with 0.01 and 0.1 μM). By contrast, the synthesis of the products < 20 nucleotides long was inhibited only 45 and 57% by 0.01 and 0.1 μM thiocoraline respectively. This may reflect a preferential inhibition of the elongation phase of the primer extension reaction by thiocoraline as opposed to inhibition of the initial stages of the reaction (e.g. initial binding of the enzyme or polymerization of the first dNTP).

### DISCUSSION

The present study shows that the novel anti-tumour drug thiocoraline, derived from the actinomycete *Micromonospora marina*, blocks cell proliferation by arresting the cells in the G1 phase of the cell cycle and decreasing the rate of S phase progression towards G2/M phases. By looking at the cell cycle perturbation by monoparametric DNA flow cytometric analysis, these effects of thiocoraline would have been missed as the block of flow of cells from G1 to S phase was compensated by the longer permanence of cells in the S phase. Instead, using biparametric flow cytometric analysis, it became evident that cells in the G1 phase during treatment remained in G1, and cells in the S phase progressed much more slowly than the control cells.

Data obtained at the National Cancer Institute showed that thiocoraline growth inhibition and cytotoxicity compares well to some other antibiotics of terrestrial origin, such as anthracyclines (e.g. daunomycin, doxorubicin). Since the mechanism of cytotoxicity of anthracyclines appears related to their ability to poison DNA Topoisomerase II enzyme we hypothesized that thiocoraline could act by the same mechanism of action and cause DNA breakage (Pommier et al, 1985; Capranico et al, 1990). However, thiocoraline did not inhibit DNA Topoisomerase II catalytic activity nor did it stimulate topoisomerase-mediated DNA cleavage in vitro. It could be argued that, in cells, the drug is metabolized to a species able to inhibit DNA Topoisomerase II. However, this was not the case, as no DNA breakage was observed in cells exposed to active thiocoraline concentrations. The good correlation existing between the pattern of sensitivity of the various cell lines in the National Institute of Health drug screen to thiocoraline and doxorubicin may be due to a common transport mechanism. Both doxorubicin and thiocoraline are good substrates for P-glycoprotein. That is, cells that were resistant to doxorubicin because of high expression of P-glycoprotein are also resistant to thiocoraline (unpublished data from this laboratory).

The cell cycle perturbations observed in cancer cells exposed to thiocoraline suggest that the drug might act by inhibiting DNA replication. Indeed, inhibition of BrdU incorporation into DNA was observed following a 24 h exposure of both LoVo and SW620 cells to thiocoraline and up to 24–48 h after drug-washout. This potent inhibitory effect of thiocoraline could be explained, at least in part, by the ability of the drug to directly inhibit DNA polymerase α activity. Primer extension by purified DNA polymerase α was almost completely inhibited by thiocoraline at concentrations that inhibited both in vitro DNA synthesis and clonogenicity. These results suggested that DNA polymerase α might be an important intracellular target for thiocoraline, although we cannot rule out that other DNA, or RNA, modifiying enzymes might also be inhibited by thiocoraline in the cells. In addition, the prolonged inhibition of DNA synthesis observed both in LoVo and SW620 cells following removal of the drug could be due to either persistence of active drug concentrations in the cells or to the induction of secondary lesions that impaired the ability of the cells to replicate DNA. Regarding the mechanism of inhibition of DNA polymerase α activity by thiocoraline, we can only speculate at present that thiocoraline may act in ways similar to other natural products (e.g. aphidicolin) that inhibit various DNA polymerases but are not structurally related to nucleoside analogues. Thiocoraline may prevent the elongation of the primers to full-length products by interacting with either the enzyme or the primer/template complex (Huberman, 1981). The predominant inhibition of the synthesis of full-length products as compared to the initiation of the primer extension reaction favour this hypothesis. This premature termination of primer extension may be due to thiocoraline-induced dissociation of DNA polymerase α from the elongating primer/template or, alternatively, 'trapping' the enzyme, presumably due to the formation of an enzyme–thiocoraline–DNA ternary complex. The latter mechanism has been proposed to explain aphidicolin-induced inhibition of DNA synthesis (Sheaff et al, 1991).

Further biochemical studies will be required to provide precise answers to these questions regarding the mechanism of inhibition of DNA polymerase activity by thiocoraline.

In theory, one could expect that a DNA synthesis inhibitor would not be very selective and would be similarly toxic against all proliferating cells. Instead, the thiocoraline concentration required to reduce the clonogenicity of SW620 cells to 50% of normal was approximately 35 times less than that required to inhibit clonogenicity of LoVo cells. These two cell lines grow at approximately the same rate, and neither LoVo nor SW620 cells express P-glycoprotein. However, they possess many biological differences, which may affect their response to thiocoraline. For example, LoVo cells express wild-type p53, whereas SW620 cells express mutated p53. As shown by biparametric flow cytometric analysis, thiocoraline induced similar cell cycle perturbations in SW620 and LoVo cells. However, a different behaviour of these two cell lines was observed following drug-washout. The G1 arrest and S phase delay were rather transient in LoVo cells, which apparently recovered within 24 h post drug incubation. In contrast, the effects of thiocoraline on the cell cycle were much more prolonged in SW620 cells, which show very little recovery even at 48 h post drug incubation. Therefore, the greater sensitivity of SW620 cells to thiocoraline was related apparently to a reduced ability of these cells to bypass the G1 and/or S phase arrest and recover from the thiocoraline-induced stress. The differential sensitivity observed in these cell lines suggests that thiocoraline is not a non-specific poison of all proliferating cells and may instead be selectively toxic for cells with some specific, but yet unknown, biological features. The determinants of the cellular sensitivity to thiocoraline and the possible role of specific genetic alterations, such as p53 mutations, are currently being investigated.

### ACKNOWLEDGEMENTS

The generous contribution of the Italian Association for Cancer Research is gratefully acknowledged.

### REFERENCES

Capranico G, Zunino F, Kohn KW and Pommier Y (1990) Sequence-selective topoisomerase II inhibition by anthracycline derivatives in SV40 DNA: relationship with DNA binding affinity and cytotoxicity. *Biochemistry* **29**: 562–569

Catapano CV, Perrino FW and Fernandes DJ (1993) Primer RNA chain termination induced by 9-beta-D-arabinofuranosyl-2-fluoroadenine 5′-triphosphate. A mechanism of DNA synthesis inhibition. *J Biol Chem* **268**: 7179–7185

Erba E, Sen S, Sessa C, Vikhanskaya FL and D'Incalci M (1994) Mechanism of cytotoxicity of 5,10-dideazatetrahydrofolic acid in human ovarian carcinoma cells in vitro and modulation of the drug activity by folic or folinic acid. *Br J Cancer* **69**: 205–211

Erba E, Mascellani E, Pifferi A and D'Incalci M (1995) Comparison of cell-cycle phase perturbations induced by the DNA-minor-groove alkylator tallimustine and by melphalan in the SW626 cell line. *Int J Cancer* **62**: 170–175

Faircloth G, Jimeno J and D'Incalci M (1997) Biological activity of thiocoraline, a novel marine depsipeptide. *Eur J Cancer* **33**: 175 (Abstract)

Faretta M, Bergamaschi D, Taverna S, Ronzoni S, Mascellani E, Cappella P, Ubezio P and Erba E (1998) Characterization of cyclin B1 expression in human cancer cell lines by a new three-parameter BrdU/cyclin B1/DNA analysis. *Cytometry* **31**: 53–59

Huberman JA (1981) New views of the biochemistry of eucaryotic DNA replication revealed by aphidicolin, an unusual inhibitor of DNA polymerase α. *Cell* **23**: 647–648

Kohn KW, Ewing RAG, Erickson LC and Zwelling LA (1981) Measurements of strand breaks and cross-links by alkaline elution. In *DNA Repair: a Laboratory Manual of Research Techniques*, Frieddberg EC and Hanawalt PC (eds). Marcel Dekker: New York

Krishan A and Frei E III (1976) Effect of adriamycin on the cell cycle traverse and kinetics of cultured human lymphoblasts. *Cancer Res* **36**: 143–150

Perez Baz J, Canedo LM, Fernandez-Puentes JL and Silva Elipe MV (1997) Thiocoraline, a novel depsipeptide with antitumor activity produced by a marine Micromonospora. II. Physico-chemical properties and structure determination. *J Antibiotics* **50**: 738–741

Poddevin B, Riou JF, Lavelle F and Pommier Y (1993) Dual topoisomerase I and II inhibition by intoplicine (RP-60475), a new antitumor agent in early clinical trials. *Mol Pharmacol* **44**: 767–774

Pommier Y, Minford JK, Schwartz RE, Zwelling LA and Kohn KW (1985) Effects of the DNA intercalators 4´-(9-acridinylamino)methanesulfon-m-anisidide and 2-methyl-9-hydroxyellipticinium on topoisomerase II mediated DNA strand cleavage and strand passage. *Biochemistry* **24**: 6410–6416

Romero F, Espliego F, Perez Baz J, Garcia de Quesada T, Gravalos D and de la Calle F (1997) Thiocoraline, a new depsipeptide with antitumor activity produced by a marine Micromonospora. I. Taxonomy, fermentation, isolation, and biological activities. *J Antibiotics* **50**: 734–737

Sheaff R, Ilsley D and Kuchta R (1991) Mechanisms of DNA polymerase alpha inhibition by aphidicolin. *Biochemistry* **30**: 8590–8597

 *© 1999 Cancer Research Campaign*
